# 4-Ethyl­phenol

**DOI:** 10.1107/S1600536809005108

**Published:** 2009-02-18

**Authors:** Richard Betz, Peter Klüfers, Peter Mayer

**Affiliations:** a Ludwig-Maximilians Universität, Department Chemie und Biochemie, Butenandtstrasse 5–13 (Haus D), 81377 München, Germany

## Abstract

The title compound, C_8_H_10_O, crystallizes with three mol­ecules in the asymmetric unit. O—H⋯O hydrogen bonds form cooperative chains connecting the mol­ecules along [100]. On the unitary graph level, this pattern is assigned a *DDD* descriptor. The ternary descriptor is *C*_3_^3^(6).

## Related literature

For the crystal structure of a co-crystallizate of the title compound and a copper complex, see: Butcher *et al.* (1995 ▶). For hydrogen-bond motifs, see: Bernstein *et al.* (1995 ▶); Etter *et al.* (1990 ▶).
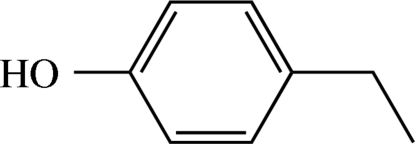


## Experimental

### 

#### Crystal data

C_8_H_10_O
*M* *_r_* = 122.16
Orthorhombic, 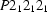

*a* = 5.9318 (19) Å
*b* = 16.514 (3) Å
*c* = 22.574 (9) Å
*V* = 2211.2 (12) Å^3^
*Z* = 12
Mo *K*α radiation
μ = 0.07 mm^−1^
*T* = 200 K
0.47 × 0.32 × 0.18 mm

#### Data collection

Oxford Diffraction Xcalibur diffractometer
Absorption correction: multi-scan (*CrysAlis RED*; Oxford Diffraction, 2005 ▶) *T*_min_ = 0.977, *T*_max_ = 0.987
9193 measured reflections
2594 independent reflections
1457 reflections with *I* > 2σ(*I*)
*R*_int_ = 0.029

#### Refinement

*R*[*F* ^2^ > 2σ(*F* ^2^)] = 0.035
*wR*(*F* ^2^) = 0.085
*S* = 0.91
2594 reflections
250 parameters
H-atom parameters constrained
Δρ_max_ = 0.13 e Å^−3^
Δρ_min_ = −0.12 e Å^−3^

### 

Data collection: *CrysAlis CCD* (Oxford Diffraction, 2005 ▶); cell refinement: *CrysAlis RED* (Oxford Diffraction, 2005 ▶); data reduction: *CrysAlis RED*; program(s) used to solve structure: *SHELXS97* (Sheldrick, 2008 ▶); program(s) used to refine structure: *SHELXL97* (Sheldrick, 2008 ▶); molecular graphics: *ORTEP-3* (Farrugia, 1997 ▶) and *Mercury* (Macrae *et al.*, 2006 ▶); software used to prepare material for publication: *SHELXL97*.

## Supplementary Material

Crystal structure: contains datablocks global, I. DOI: 10.1107/S1600536809005108/jh2071sup1.cif

Structure factors: contains datablocks I. DOI: 10.1107/S1600536809005108/jh2071Isup2.hkl

Additional supplementary materials:  crystallographic information; 3D view; checkCIF report

## Figures and Tables

**Table 1:** Hydrogen-bond geometry (Å, °)

*D*—H⋯*A*	*D*—H	H⋯*A*	*D*⋯*A*	*D*—H⋯*A*
O1—H1⋯O3^i^	0.84	1.84	2.662 (2)	166
O2—H2⋯O1^ii^	0.84	1.81	2.642 (2)	171
O3—H3⋯O2^iii^	0.84	1.84	2.664 (2)	165

Symmetry codes: (i) 
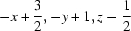
; (ii) 
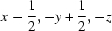
; (iii) 
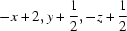
.

**Fig. 1.**
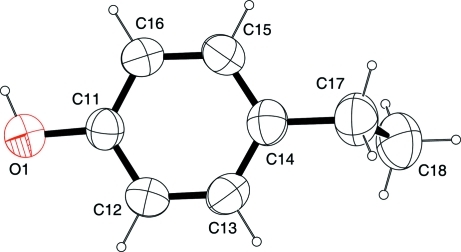
The molecular structure of one molecule in the asymmetric unit of the title compound, with atom labels and anisotropic displacement ellipsoids (drawn at 50% probability level) for non-H atoms.

**Fig. 2.**
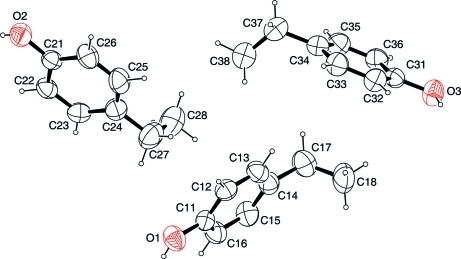
The asymmetric unit of the title compound, with atom labels and anisotropic displacement ellipsoids (drawn at 50% probability level) for non-H atoms.

**Fig. 3.**
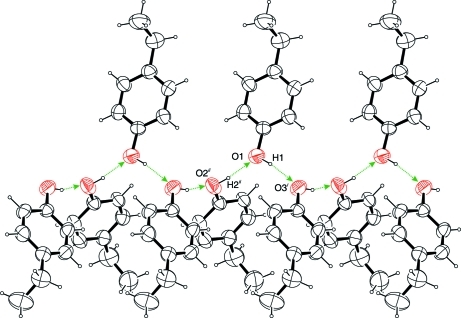
Hydrogen bonds in the crystal structure of the title compound, viewed along [0 1 0]. Symmetry operators: i -x + 3/2, -y + 1, z - 1/2; iix + 1/2, -y + 1/2, -z.

**Fig. 4.**
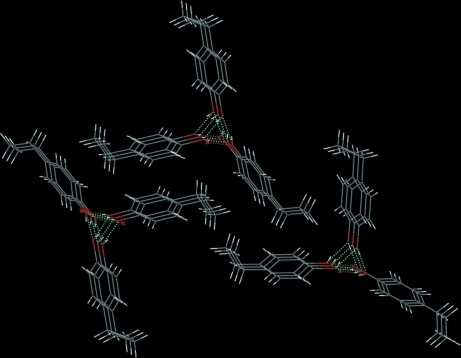
The pseudo-trigonal packing of the molecules in the crystal structure, viewed approximately along [1 0 0].

**Fig. 5.**
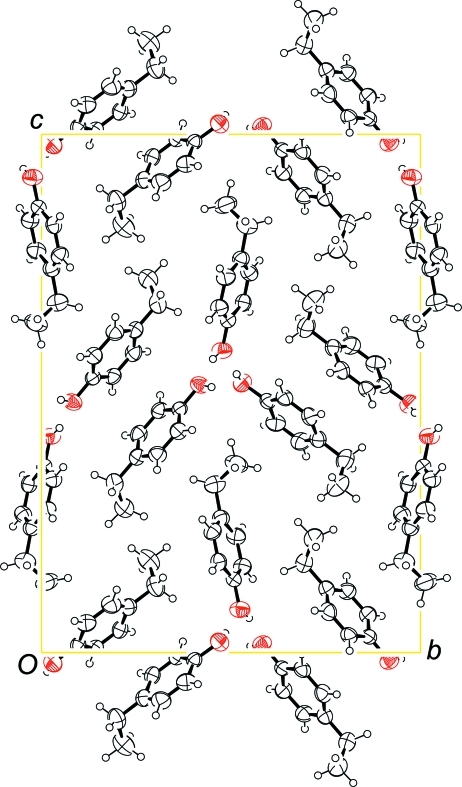
The packing of the title compound, viewed along [-1 0 0].

## supplementary crystallographic information

### Comment

In a program focused on the influence of bonding to pentavalent central atoms on the geometry of aromatic alcohols, the crystal structure of *para*-ethylphenol was determined.

There are three molecules in the asymmetric unit which do not show non-crystallographic symmetry (Fig. 1 and Fig. 2). The hydrogen atoms on the hydroxy groups reside in the planes of the respective aromatic moiety whereas the alkyl chains are oriented approximately perpendicular to them. The least-squares planes defined by the aromatic moieties and the atoms of the hydroxy group and their respective aromatic carrier atom enclose angles roughly between 1° and 16°. The least-squares planes defined by the aromatic moieties and the C atoms of the alkyl chain and their respective aromatic carrier atom intersect at angles roughly between 77° and 80°.

The hyxdroxy groups furnish the formation of cooperative chains of hydrogen bonds along [1 0 0] (Fig. 3). In terms of graph-set analysis (Etter *et al.*, 1990; Bernstein *et al.*, 1995), this pattern can be ascribed a *DDD* descriptor on the unitary level. The ternary descriptor of these chains is *C*_3_^3^(6). The molecules are packed in a pseudo-trigonal mode with the hydrophilic part of the molecules residing in the center of these entities (Fig. 4).

The molecular packing of the title compound is shown in Figure 5.

### Experimental

The compound was obtained commercially (Aldrich) and used for diffraction studies as received.

### Refinement

Due to the absence of a strong anomalous scatterer in the molecule the absolute structure parameter, which is 0.7948 with an estimated standard deviation of 1.2829 for the unmerged data set, is meaningless. Thus, Friedel opposites (1849 pairs) have been merged and the absolute configuration has been arbitrarily chosen. The absolute structure parameter has been removed from the cif.

Carbon-bound H-atoms were placed in calculated positions (C—H 0.95 Å for aromatic C atoms, C—H 0.99 Å for methylene groups, C—H 0.98 Å for methyl groups and O—H 0.84 Å) and were included in the refinement in the riding model approximation, with *U*(H) set to 1.2*U*_eq_(C) for aromatic C atoms and methylene groups, *U*(H) set to 1.5*U*_eq_(C) for methyl groups and *U*(H) set to 1.5*U*_eq_(O) for the hydroxy groups.

### Crystal data

**Table:**

C_8_H_10_O	*F*(000) = 792
*M**_r_* = 122.16	*D*_x_ = 1.101 Mg m^−^^3^
Orthorhombic, *P*2_1_2_1_2_1_	Mo *K*α radiation, λ = 0.71073 Å
Hall symbol: P 2ac 2ab	Cell parameters from 3039 reflections
*a* = 5.9318 (19) Å	θ = 4.1–26.3°
*b* = 16.514 (3) Å	µ = 0.07 mm^−^^1^
*c* = 22.574 (9) Å	*T* = 200 K
*V* = 2211.2 (12) Å^3^	Block, colourless
*Z* = 12	0.47 × 0.32 × 0.18 mm

### Data collection

**Table:**

Oxford Diffraction Xcalibur diffractometer	2594 independent reflections
Radiation source: fine-focus sealed tube	1457 reflections with *I* > 2σ(*I*)
graphite	*R*_int_ = 0.029
ω scans	θ_max_ = 26.3°, θ_min_ = 4.1°
Absorption correction: multi-scan (*CrysAlis RED*; Oxford Diffraction, 2005)	*h* = −7→7
*T*_min_ = 0.977, *T*_max_ = 0.987	*k* = −20→20
9193 measured reflections	*l* = −28→8

### Refinement

**Table:**

Refinement on *F*^2^	Primary atom site location: structure-invariant direct methods
Least-squares matrix: full	Secondary atom site location: difference Fourier map
*R*[*F*^2^ > 2σ(*F*^2^)] = 0.035	Hydrogen site location: inferred from neighbouring sites
*wR*(*F*^2^) = 0.085	H-atom parameters constrained
*S* = 0.91	*w* = 1/[σ^2^(*F*_o_^2^) + (0.0451*P*)^2^] where *P* = (*F*_o_^2^ + 2*F*_c_^2^)/3
2594 reflections	(Δ/σ)_max_ < 0.001
250 parameters	Δρ_max_ = 0.13 e Å^−^^3^
0 restraints	Δρ_min_ = −0.12 e Å^−^^3^

### Special details

**Table:**

Experimental. CrysAlis RED, Oxford Diffraction Ltd., Version 1.171.32.5 (release 08-05-2007 CrysAlis171 .NET) (compiled May 8 2007,13:10:02) Empirical absorption correction using spherical harmonics, implemented in SCALE3 ABSPACK scaling algorithm.

### Fractional atomic coordinates and isotropic or equivalent isotropic displacement parameters (Å^2^)

**Table:**

	*x*	*y*	*z*	*U*_iso_*/*U*_eq_	
O1	0.8108 (3)	0.52361 (10)	0.08295 (8)	0.0654 (5)	
H1	0.7025	0.5476	0.0666	0.098*	
C11	0.7567 (4)	0.50737 (12)	0.14108 (12)	0.0477 (6)	
C12	0.9136 (4)	0.46767 (13)	0.17501 (12)	0.0526 (6)	
H12	1.0528	0.4513	0.1580	0.063*	
C13	0.8699 (4)	0.45164 (13)	0.23352 (12)	0.0564 (7)	
H13	0.9806	0.4248	0.2567	0.068*	
C14	0.6677 (4)	0.47378 (13)	0.25946 (11)	0.0550 (6)	
C15	0.5121 (4)	0.51333 (14)	0.22438 (12)	0.0596 (7)	
H15	0.3722	0.5293	0.2412	0.072*	
C16	0.5542 (4)	0.53024 (14)	0.16553 (12)	0.0561 (6)	
H16	0.4445	0.5574	0.1422	0.067*	
C17	0.6225 (5)	0.45737 (16)	0.32442 (12)	0.0761 (8)	
H171	0.4586	0.4618	0.3320	0.091*	
H172	0.6691	0.4013	0.3339	0.091*	
C18	0.7444 (6)	0.51441 (17)	0.36405 (14)	0.0893 (10)	
H181	0.9069	0.5104	0.3567	0.134*	
H182	0.7129	0.5006	0.4054	0.134*	
H183	0.6939	0.5699	0.3561	0.134*	
O2	0.6558 (3)	0.03018 (10)	−0.02023 (8)	0.0633 (5)	
H2	0.5370	0.0151	−0.0372	0.095*	
C21	0.6100 (4)	0.09676 (13)	0.01496 (11)	0.0475 (6)	
C22	0.4167 (4)	0.14128 (13)	0.00819 (11)	0.0517 (6)	
H22	0.3093	0.1269	−0.0212	0.062*	
C23	0.3797 (4)	0.20736 (13)	0.04461 (11)	0.0544 (6)	
H23	0.2457	0.2382	0.0398	0.065*	
C24	0.5336 (5)	0.22977 (13)	0.08795 (11)	0.0559 (6)	
C25	0.7266 (4)	0.18400 (16)	0.09256 (12)	0.0652 (7)	
H25	0.8362	0.1984	0.1214	0.078*	
C26	0.7663 (4)	0.11799 (15)	0.05676 (11)	0.0603 (7)	
H26	0.9010	0.0874	0.0611	0.072*	
C27	0.4897 (6)	0.30142 (15)	0.12800 (13)	0.0811 (9)	
H271	0.6348	0.3204	0.1446	0.097*	
H272	0.4253	0.3461	0.1041	0.097*	
C28	0.3328 (5)	0.28252 (17)	0.17770 (14)	0.0902 (10)	
H281	0.1855	0.2669	0.1617	0.135*	
H282	0.3155	0.3304	0.2029	0.135*	
H283	0.3942	0.2378	0.2012	0.135*	
O3	1.0222 (3)	0.41786 (9)	0.51646 (7)	0.0624 (5)	
H3	1.1336	0.4488	0.5123	0.094*	
C31	1.0136 (4)	0.36433 (13)	0.46965 (10)	0.0470 (6)	
C32	1.1787 (4)	0.36152 (13)	0.42740 (11)	0.0540 (6)	
H32	1.3032	0.3976	0.4295	0.065*	
C33	1.1639 (4)	0.30624 (14)	0.38184 (11)	0.0580 (7)	
H33	1.2791	0.3051	0.3526	0.070*	
C34	0.9863 (4)	0.25252 (13)	0.37750 (11)	0.0537 (6)	
C35	0.8229 (4)	0.25701 (14)	0.42103 (12)	0.0593 (7)	
H35	0.6984	0.2210	0.4191	0.071*	
C36	0.8333 (4)	0.31177 (13)	0.46722 (11)	0.0537 (6)	
H36	0.7189	0.3132	0.4966	0.064*	
C37	0.9689 (5)	0.19321 (15)	0.32700 (13)	0.0732 (8)	
H371	1.1204	0.1858	0.3094	0.088*	
H372	0.9198	0.1402	0.3429	0.088*	
C38	0.8098 (6)	0.21842 (17)	0.27951 (14)	0.0975 (11)	
H381	0.6581	0.2244	0.2962	0.146*	
H382	0.8074	0.1772	0.2483	0.146*	
H383	0.8589	0.2703	0.2628	0.146*	

### Atomic displacement parameters (Å^2^)

**Table:**

	*U*^11^	*U*^22^	*U*^33^	*U*^12^	*U*^13^	*U*^23^
O1	0.0630 (11)	0.0822 (12)	0.0510 (12)	0.0136 (9)	0.0028 (10)	0.0007 (9)
C11	0.0533 (14)	0.0462 (13)	0.0436 (15)	−0.0010 (11)	−0.0005 (14)	−0.0036 (12)
C12	0.0483 (14)	0.0503 (12)	0.0592 (17)	0.0030 (12)	0.0019 (14)	−0.0025 (13)
C13	0.0525 (16)	0.0599 (14)	0.0568 (18)	0.0011 (12)	−0.0090 (14)	0.0066 (13)
C14	0.0585 (16)	0.0567 (13)	0.0499 (17)	−0.0093 (14)	−0.0015 (15)	−0.0014 (13)
C15	0.0496 (14)	0.0723 (16)	0.0570 (18)	0.0053 (13)	0.0067 (14)	−0.0043 (14)
C16	0.0502 (15)	0.0632 (14)	0.0549 (18)	0.0086 (13)	−0.0040 (14)	0.0009 (13)
C17	0.0816 (19)	0.0873 (18)	0.0593 (19)	−0.0029 (16)	0.0041 (17)	0.0073 (16)
C18	0.107 (2)	0.103 (2)	0.0581 (19)	0.0122 (19)	−0.0030 (19)	−0.0198 (17)
O2	0.0573 (10)	0.0672 (10)	0.0655 (12)	0.0142 (9)	−0.0156 (9)	−0.0082 (9)
C21	0.0473 (14)	0.0467 (12)	0.0484 (15)	0.0009 (12)	−0.0050 (13)	0.0052 (12)
C22	0.0460 (14)	0.0564 (13)	0.0527 (16)	0.0001 (12)	−0.0121 (12)	0.0067 (13)
C23	0.0507 (14)	0.0492 (13)	0.0634 (17)	0.0051 (12)	−0.0008 (14)	0.0063 (13)
C24	0.0579 (16)	0.0531 (13)	0.0566 (17)	−0.0128 (13)	−0.0007 (15)	0.0028 (12)
C25	0.0577 (16)	0.0768 (17)	0.0610 (19)	−0.0112 (14)	−0.0163 (15)	−0.0031 (16)
C26	0.0467 (15)	0.0769 (17)	0.0574 (17)	0.0039 (12)	−0.0119 (14)	0.0031 (15)
C27	0.093 (2)	0.0643 (16)	0.086 (2)	−0.0161 (16)	−0.004 (2)	−0.0143 (16)
C28	0.083 (2)	0.095 (2)	0.093 (2)	0.0042 (17)	0.004 (2)	−0.0303 (19)
O3	0.0623 (11)	0.0705 (11)	0.0543 (11)	−0.0142 (9)	0.0107 (10)	−0.0089 (9)
C31	0.0506 (13)	0.0472 (12)	0.0433 (15)	−0.0024 (12)	0.0003 (13)	0.0039 (11)
C32	0.0473 (13)	0.0573 (13)	0.0576 (17)	−0.0096 (12)	0.0090 (14)	−0.0003 (14)
C33	0.0556 (15)	0.0645 (15)	0.0539 (18)	0.0014 (14)	0.0101 (13)	−0.0001 (13)
C34	0.0566 (14)	0.0490 (13)	0.0556 (17)	0.0033 (13)	−0.0017 (15)	0.0041 (12)
C35	0.0573 (15)	0.0549 (14)	0.0657 (19)	−0.0151 (12)	−0.0046 (16)	0.0063 (14)
C36	0.0531 (14)	0.0563 (14)	0.0517 (17)	−0.0103 (13)	0.0086 (13)	0.0076 (13)
C37	0.088 (2)	0.0570 (14)	0.074 (2)	−0.0032 (15)	−0.0042 (18)	−0.0060 (15)
C38	0.144 (3)	0.0730 (18)	0.076 (2)	0.001 (2)	−0.022 (2)	−0.0089 (16)

### Geometric parameters (Å, °)

**Table:**

O1—C11	1.377 (3)	C25—C26	1.377 (3)
O1—H1	0.8400	C25—H25	0.9500
C11—C12	1.372 (3)	C26—H26	0.9500
C11—C16	1.375 (3)	C27—C28	1.491 (4)
C12—C13	1.372 (3)	C27—H271	0.9900
C12—H12	0.9500	C27—H272	0.9900
C13—C14	1.384 (3)	C28—H281	0.9800
C13—H13	0.9500	C28—H282	0.9800
C14—C15	1.380 (3)	C28—H283	0.9800
C14—C17	1.515 (4)	O3—C31	1.379 (2)
C15—C16	1.380 (3)	O3—H3	0.8400
C15—H15	0.9500	C31—C32	1.368 (3)
C16—H16	0.9500	C31—C36	1.378 (3)
C17—C18	1.487 (4)	C32—C33	1.378 (3)
C17—H171	0.9900	C32—H32	0.9500
C17—H172	0.9900	C33—C34	1.381 (3)
C18—H181	0.9800	C33—H33	0.9500
C18—H182	0.9800	C34—C35	1.382 (3)
C18—H183	0.9800	C34—C37	1.506 (3)
O2—C21	1.383 (3)	C35—C36	1.382 (3)
O2—H2	0.8400	C35—H35	0.9500
C21—C26	1.368 (3)	C36—H36	0.9500
C21—C22	1.371 (3)	C37—C38	1.488 (4)
C22—C23	1.384 (3)	C37—H371	0.9900
C22—H22	0.9500	C37—H372	0.9900
C23—C24	1.388 (3)	C38—H381	0.9800
C23—H23	0.9500	C38—H382	0.9800
C24—C25	1.376 (4)	C38—H383	0.9800
C24—C27	1.512 (3)		
			
C11—O1—H1	109.5	C21—C26—C25	119.5 (2)
C12—C11—C16	120.0 (2)	C21—C26—H26	120.3
C12—C11—O1	117.8 (2)	C25—C26—H26	120.3
C16—C11—O1	122.2 (2)	C28—C27—C24	113.2 (2)
C11—C12—C13	120.1 (2)	C28—C27—H271	108.9
C11—C12—H12	120.0	C24—C27—H271	108.9
C13—C12—H12	120.0	C28—C27—H272	108.9
C12—C13—C14	121.4 (2)	C24—C27—H272	108.9
C12—C13—H13	119.3	H271—C27—H272	107.8
C14—C13—H13	119.3	C27—C28—H281	109.5
C15—C14—C13	117.5 (2)	C27—C28—H282	109.5
C15—C14—C17	121.4 (2)	H281—C28—H282	109.5
C13—C14—C17	121.0 (2)	C27—C28—H283	109.5
C14—C15—C16	121.8 (2)	H281—C28—H283	109.5
C14—C15—H15	119.1	H282—C28—H283	109.5
C16—C15—H15	119.1	C31—O3—H3	109.5
C11—C16—C15	119.3 (2)	C32—C31—O3	122.0 (2)
C11—C16—H16	120.4	C32—C31—C36	120.4 (2)
C15—C16—H16	120.4	O3—C31—C36	117.6 (2)
C18—C17—C14	112.5 (2)	C33—C32—C31	119.8 (2)
C18—C17—H171	109.1	C33—C32—H32	120.1
C14—C17—H171	109.1	C31—C32—H32	120.1
C18—C17—H172	109.1	C32—C33—C34	121.8 (2)
C14—C17—H172	109.1	C32—C33—H33	119.1
H171—C17—H172	107.8	C34—C33—H33	119.1
C17—C18—H181	109.5	C35—C34—C33	116.7 (2)
C17—C18—H182	109.5	C35—C34—C37	121.7 (2)
H181—C18—H182	109.5	C33—C34—C37	121.6 (2)
C17—C18—H183	109.5	C36—C35—C34	122.7 (2)
H181—C18—H183	109.5	C36—C35—H35	118.6
H182—C18—H183	109.5	C34—C35—H35	118.6
C21—O2—H2	109.5	C35—C36—C31	118.5 (2)
C26—C21—C22	120.4 (2)	C35—C36—H36	120.8
C26—C21—O2	117.8 (2)	C31—C36—H36	120.8
C22—C21—O2	121.8 (2)	C38—C37—C34	114.0 (2)
C21—C22—C23	119.3 (2)	C38—C37—H371	108.8
C21—C22—H22	120.3	C34—C37—H371	108.8
C23—C22—H22	120.3	C38—C37—H372	108.8
C22—C23—C24	121.6 (2)	C34—C37—H372	108.8
C22—C23—H23	119.2	H371—C37—H372	107.6
C24—C23—H23	119.2	C37—C38—H381	109.5
C25—C24—C23	117.0 (2)	C37—C38—H382	109.5
C25—C24—C27	121.9 (3)	H381—C38—H382	109.5
C23—C24—C27	121.1 (2)	C37—C38—H383	109.5
C24—C25—C26	122.2 (2)	H381—C38—H383	109.5
C24—C25—H25	118.9	H382—C38—H383	109.5
C26—C25—H25	118.9		
			
C16—C11—C12—C13	−0.8 (3)	C27—C24—C25—C26	179.1 (2)
O1—C11—C12—C13	178.6 (2)	C22—C21—C26—C25	0.8 (4)
C11—C12—C13—C14	0.9 (3)	O2—C21—C26—C25	179.9 (2)
C12—C13—C14—C15	−0.6 (3)	C24—C25—C26—C21	0.2 (4)
C12—C13—C14—C17	−178.8 (2)	C25—C24—C27—C28	−100.3 (3)
C13—C14—C15—C16	0.2 (3)	C23—C24—C27—C28	79.8 (3)
C17—C14—C15—C16	178.4 (2)	O3—C31—C32—C33	179.2 (2)
C12—C11—C16—C15	0.5 (3)	C36—C31—C32—C33	0.5 (3)
O1—C11—C16—C15	−178.9 (2)	C31—C32—C33—C34	−0.4 (3)
C14—C15—C16—C11	−0.2 (3)	C32—C33—C34—C35	0.2 (3)
C15—C14—C17—C18	−102.5 (3)	C32—C33—C34—C37	178.9 (2)
C13—C14—C17—C18	75.6 (3)	C33—C34—C35—C36	−0.3 (3)
C26—C21—C22—C23	−0.9 (3)	C37—C34—C35—C36	−178.9 (2)
O2—C21—C22—C23	−179.9 (2)	C34—C35—C36—C31	0.4 (3)
C21—C22—C23—C24	0.0 (3)	C32—C31—C36—C35	−0.5 (3)
C22—C23—C24—C25	0.9 (3)	O3—C31—C36—C35	−179.27 (19)
C22—C23—C24—C27	−179.2 (2)	C35—C34—C37—C38	78.0 (3)
C23—C24—C25—C26	−1.0 (4)	C33—C34—C37—C38	−100.6 (3)

### Hydrogen-bond geometry (Å, °)

**Table:**

*D*—H···*A*	*D*—H	H···*A*	*D*···*A*	*D*—H···*A*
O1—H1···O3^i^	0.84	1.84	2.662 (2)	166
O2—H2···O1^ii^	0.84	1.81	2.642 (2)	171
O3—H3···O2^iii^	0.84	1.84	2.664 (2)	165

Symmetry codes: (i) −*x*+3/2, −*y*+1, *z*−1/2; (ii) *x*−1/2, −*y*+1/2, −*z*; (iii) −*x*+2, *y*+1/2, −*z*+1/2.
